# Describe or Direct? Artificial Intelligence Explanation Type, Trust and User Behaviour in a Medical Imaging Rostering Task

**DOI:** 10.1002/jmrs.70137

**Published:** 2026-10-20

**Authors:** Kait O'Callahan, Sharon Sitters, Michael O'Callahan, Adam Senior‐Partridge, Max Kirtley

**Affiliations:** ^1^ Unitec Auckland New Zealand

## Abstract

**Introduction:**

Artificial intelligence (AI) is increasingly integrated into healthcare, with most healthcare AI applications concentrated in radiology. While explainable AI (XAI) has been proposed as a means of improving transparency and trust, the relationship between explanation features, trust and user behaviour remains underexplored, particularly in healthcare contexts. This study examined how final‐year medical imaging students interacted with descriptive and prescriptive AI explanations during a simulated rostering task, with a focus on trust and user behaviour.

**Methods:**

A human‐grounded evaluation using an explanatory mixed methods design was undertaken. Final year medical imaging students completed simulated rostering tasks using an XAI system that generated either descriptive or prescriptive explanations. Quantitative measures collected included task completion metrics, explanation regenerations and self‐reported trust scores. Participants took part in semi‐structured discussion groups to explore user perceptions, explanation preferences and trust dynamics.

**Results:**

Trust scores increased across the experiment for both explanation types. Prescriptive explanations were associated with significantly fewer explanation regenerations and higher trust scores. Task time and failed solutions showed small‐to‐moderate effects favouring prescriptive explanations, but these differences did not reach statistical significance. Qualitative findings indicated a strong participant preference for prescriptive explanations, alongside critical engagement with inaccuracies and regeneration patterns.

**Conclusion:**

Prescriptive AI explanations were preferred over descriptive explanations within a simulated medical imaging rostering task and were associated with higher trust and reduced explanation regeneration. Participants demonstrated critical engagement with AI‐generated outputs rather than uncritical acceptance. These findings contribute to emerging evidence regarding human interaction with XAI systems in healthcare workflow settings.

## Graphical abstract

This study explored how descriptive and prescriptive AI explanations influenced trust and user behaviour among 20 final‐year medical imaging students completing 20 realistic rostering tasks. Prescriptive explanations were preferred and associated with higher trust and fewer explanation regenerations, while task time and failed solutions did not differ significantly between explanation types. The graphical abstract was generated using ChatGPT (OpenAI, GP‐T5.6 Luna).

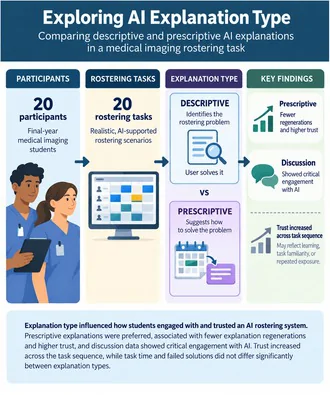

## 1 Introduction

### 1.1 The Role of Explainable AI in Healthcare

Artificial intelligence (AI) is rapidly advancing and increasingly integrated into healthcare settings [1], with a majority of AI technologies for healthcare in radiology departments [2]. Although AI can outperform humans in certain analytical tasks, its lack of explainability has drawn criticism [3] and led to mistrust [4]. This, in turn, can reduce user engagement [5]. As AI systems become more complex, they often function as ‘black boxes’, obscuring the rationale behind their outputs. This opacity presents significant barriers to trust, particularly when compared to traditional decision‐making processes [6].

As a result, there is a growing need for healthcare workers and other stakeholders to better understand how AI makes decisions. Explainable AI (XAI) offers a potential solution. Explainability refers to an AI system's ability to communicate the factors that contributed to a specific decision [4]. However, where present, current methods for providing XAI often ignore the human user in the design process [7]. Given the rapid expansion of AI technologies in healthcare, and the associated challenges of implementation and change management, the development of human‐centred XAI is essential to realise the full benefits of AI for healthcare improvement [6, 8, 9].

### 1.2 Trust and Explainability in AI Systems

Trust in technology has been defined as ‘the attitude that an agent will help achieve an individual's goals in a situation characterized by uncertainty and vulnerability’ [10]. While there is general agreement in the literature that XAI features should promote appropriate user trust [4], the relationship between XAI and trust is complex. XAI can increase trust [4], encourage critical thinking [11] or in some cases reduce trust [12]. Both insufficient and excessive trust are problematic; low trust may result in underuse of beneficial systems [13], while over trust can lead to automation bias and error [14]. Therefore, establishing appropriate levels of trust through explainability is essential. Authors have sought to understand how varying mental models express aspects of trust in AI [15], how trust develops longitudinally and how it evolves through interaction [16]; however, no trust model is specifically tailored to contexts involving human‐impact decisions or healthcare settings.

Ideally, XAI enables users to trust AI when justified while remaining critical and engaged. This is only possible if explanations are understandable, allowing users to evaluate the reasoning behind a decision [17]. Trust among healthcare professionals is critical to the successful integration and effective use of AI [18].

### 1.3 Explainable AI in Healthcare Rostering

AI‐generated rostering shows promise in reducing administrative burden and improving work‐life balance for healthcare workers. Traditionally, medical imaging rostering is done by a senior member of staff, taking a significant amount of time away from clinical duties. AI‐generated rostering therefore has potential to reduce the administrative burden associated with workforce allocation while allowing senior clinical staff to spend more time on clinical and operational responsibilities [19]. However, resistance to AI can arise when users do not understand how decisions are made or find system outputs confusing [19]. In the rostering context, better explainability may foster greater trust, support critical assessment of decisions and improve problem‐solving, ultimately leading to more efficient rostering.

A key challenge for developers is ensuring explanations are comprehensible to end users [20]. Large language models (LLMs) show promise in solving this issue, as they can generate text that convincingly mimics that of a human [21]. However, LLMs can produce incorrect outputs commonly referred to as hallucinations, requiring user verification in domains where accuracy is critical. LLMs may embed hallucinations with otherwise accurate statements, making them difficult to detect [22]. Furthermore, generating explanations is not enough; it is imperative these explanations are evaluated for clarity and effectiveness [16]. Therefore, XAI features should be developed in collaboration with users. This study explores these issues by comparing explanation types in a rostering context. The XAI features chosen for this study were descriptive and prescriptive explanations provided to final‐year medical imaging students during a rostering task. This comparison was chosen to evaluate practical usability in a healthcare rostering context, specifically whether students preferred identification of problems with autonomy to solve them, or direct guidance towards a solution. AI systems that suggest solutions should balance passive trust with mechanisms that support critical thinking [23]. Descriptive explanations do not offer solutions, requiring users to generate their own solution, which may reduce the risk of over‐reliance. This is consistent with the principle that most modern AI systems are designed to support, rather than replace, human decision‐making [23]. Descriptive explanations are more constrained and therefore involve fewer generated claims and lower exposure to error. However, prescriptive explanations may be more suitable for action‐driven tasks.

This study investigates:

1. Whether prescriptive or descriptive explanations are preferred by medical imaging students.
2. Whether explanation type influences explanation regeneration, task completion time and failed solutions.
3. Whether explanation type influences self‐reported trust, and how participants describe their experiences and responses to the XAI system.

The overall aim was to compare descriptive and prescriptive explanation types and explore how users responded to these approaches in a medical imaging rostering context.

## 2 Methods

### 2.1 Human‐Grounded Evaluation

This study used a human‐grounded evaluation approach [17] to explore trust development when using explainable AI (XAI) to solve rostering problems. A simplified experiment was conducted in which participants solved realistic rostering tasks typical of AI‐powered medical imaging rosters [17]. This method allows testing of multiple explanations without the need for extensive development of deployed systems. Papagni et al. [24] suggest that explainees in XAI contexts are often treated as novice users, with systems designed under the assumption of limited prior domain knowledge.

For this reason, final‐year medical imaging students were chosen as participants; they represent novice users with respect to rostering systems. While they are familiar with clinical workflows and general rostering patterns, no formal experience with rostering within their current training context or with Rosterlab systems was expected; therefore, reducing the influence of prior domain heuristics as a confounding factor.

### 2.2 Participant Information

Twenty final‐year medical imaging students were recruited via convenience sampling. This sample was selected due to accessibility and their limited prior exposure to formal rostering systems. Participants had substantial clinical experience through approximately 43 weeks of clinical placements and were near transition to professional practice (approximately 10 clinical weeks from completion), providing familiarity with healthcare environments and routine clinical workflows. This group was approaching graduate practice with relevant healthcare exposure but limited rostering expertise, making them suitable for evaluating explanation‐driven responses to AI‐generated rostering tasks. As an exploratory study, the sample was not intended to represent experienced radiographers or rostering staff and findings may differ in populations with greater rostering or clinical experience.

Participant selection was based on a first‐in policy. Ethics approval was obtained from the Unitec Research Ethics committee (application number 2024‐1049), and informed consent was obtained from all participants. The study was supported by internal institutional funding. Participants received a gift voucher for their time.

### 2.3 XAI Feature Design

Explanatory features were developed in collaboration with an AI‐generated self‐rostering company (Rosterlab). A LLM was used to translate algorithmic rostering outputs into explanations for XAI purposes. The rostering task was framed around real‐world infeasibility problems, with outputs presented in two conditions: (a) *descriptive* or (b) *prescriptive*. In this study, we adopted the descriptive vs. prescriptive definition from analytics literature [25], where descriptive features present what the model has identified, while prescriptive features suggest potential actions based on the model's output.

### 2.4 Prompt Generation

Explanatory outputs were generated using a LLM (Claude, Anthropic; Sonnet 3.5). Prompts were developed using a structured template to ensure consistency across tasks and conditions.

Each prompt consisted of three components: (1) a static segment defining the rostering environment and constraints, including staff roles, shift requirements and operational rules; (2) a scenario‐specific statement describing a rostering infeasibility; and (3) an instruction determining the explanation type. The static segment remained constant across all prompts, while the scenario content varied to reflect different problem types and levels of complexity.

The final instruction in each prompt determined whether the model produced a descriptive or prescriptive explanation and was the only element that differed between prompt conditions. In the descriptive condition, the model was instructed to identify and describe issues within the roster without suggesting solutions. In the prescriptive condition, the model was instructed to propose actionable solutions to resolve the identified issues. Aside from this final instruction, prompts per rostering problem were identical across conditions.

Prompts were manually developed and iteratively refined using real‐world rostering constraints provided by Rosterlab. Outputs were reviewed 20 times each per prompt during development to ensure clarity, relevance and alignment with the intended explanation type. No post‐processing or filtering of model outputs was applied. Regenerated outputs were produced using identical prompts, such that prompt content remained constant across generations.

During the experiment, outputs were not independently validated for factual accuracy following generation. Consequently, participant‐identified inaccuracies and hallucinations formed part of the user interaction being evaluated rather than being removed from the study. Because regenerated outputs were produced using identical prompts, variation between generations also formed part of the interaction under investigation, although this variability was not independently quantified.

The static prompt templates, an example rostering problem and example outputs are provided in Supporting Information (Appendix A).

### 2.5 Experiment Design

Participants attended an introductory session and received a basic overview of the Rosterlab interface. Each was assigned a computer in a lab and provided with a walk‐through document outlining the steps for the experiment (e.g., force roster failure, generate an explanation, solve the rostering problem, complete the survey). Researchers were present on site to provide support as needed.

Participants individually worked through 20 rostering tasks (labelled as problems in the Results based on industry terminology) in a fixed sequence. They completed tasks using two explanation types: descriptive (*D*) and prescriptive (*P*). Explanation type was counterbalanced across participants. Participants S01–S10 received descriptive explanations for odd‐numbered tasks and prescriptive explanations for even‐numbered tasks, while participants S11–S20 received the reverse sequence (Supporting Information, Appendix B). Participants were encouraged to spend no more than 30 min per task but could skip and move on if they became stuck. The timestamp and result (failed or solved) of all solutions were recorded, along with the timestamp and type (*D* or *P*) of all explanations generated.

### 2.6 Rostering Task Design

Rostering tasks were provided by Rosterlab and based on real‐world challenges encountered by their users. These tasks incorporated constraints related to local union agreements, medical imaging department requirements, staffing demands, among other factors. Hypothetical staff were given familiar New Zealand names to enhance realism. All tasks were solved inside the Rosterlab interface, with the Claude API providing the XAI feature separately (Supporting Information, Appendix B).

### 2.7 Quantitative Data

Collected data included the provided solution type, time spent solving each task and the result (solved or not solved), the number of generations and the average count of failed solves paired by user. After each task, participants completed a Google Forms survey of various aspects of trust, regardless of whether the problem was solved.

### 2.8 Survey Design

The seven item survey was adapted from existing instruments [26, 27, 28], based on the modified Cahour‐Fourzy scale, presented by Hoffman et al. [16, 29]; where Item 5 was adapted from the Jian et al. Scale [26], Item 6 was adapted from the Schaefer Scale [27] and Item 7 was adapted from the Madsen‐Gregor Scale [28]. Although the reliability of the Recommended Scale has not been independently validated, its substantial overlap with the Jian et al. scale [26] and its semantic similarity to the Madsen‐Gregor Scale [28], both of which demonstrated high reliability, suggest that the Recommended Scale may also exhibit acceptable reliability. While these findings support the potential reliability of the Recommended Scale, formal reliability assessment was not performed. A study‐specific Trust score was calculated as the mean of Confidence, Reliability, Safety, Efficiency and Likes AI, as these items were considered to represent participants' overall positive assessment of the XAI system. Wariness and Human Preference were analysed separately and were not included in the Trust score. No independent reliability testing was undertaken because the measure was adapted for this exploratory study rather than used as a validated psychometric scale.

### 2.9 Survey Analysis

Survey data were collected across seven domains: Confidence, Reliability, Safety, Efficiency, Wariness, Human Preferences and Likes AI. From these measures, two composite variables were created. A Trust score was computed as the mean of Confidence, Reliability, Safety, Efficiency and Likes AI. Analysis consisted of calculating the mean trust scores for each question and then visualising results with line graphs.

### 2.10 Performance Analysis

Normality of paired differences between conditions D and P was assessed using the Shapiro–Wilk test for each outcome measure. If the differences were approximately normally distributed, a paired *t*‐test was applied. If normality was questionable, the Wilcoxon signed‐rank test was used either as a robustness check or as the primary test.

Effect sizes were calculated to quantify the magnitude of observed differences. For parametric tests, Cohen's *d*_z_ was computed for paired samples; for non‐parametric tests, the effect size *r* was derived from the Wilcoxon signed‐rank statistic.

Exact *p*‐values and effect sizes were reported for all paired comparisons, with 95% confidence intervals for mean paired differences where appropriate. No adjustment for multiple comparisons was applied because the analyses were exploratory; findings were interpreted with consideration of effect sizes and the number of comparisons undertaken.

All statistical analyses and visualisations were conducted in Python (version 3.12.3) using the pandas, scipy.stats, numpy and matplotlib libraries.

### 2.11 Discussion Groups

An explanatory sequential design was utilised, whereby discussion group data was used to explore observed patterns in trust. A total of four discussion groups were conducted, with five participants in each group. Groups lasted approximately 45 min and were conducted as semi‐structured discussions using questions relating to explanation preference, trust, regeneration and user responses.

Discussion group data was transcribed and analysed deductively using a structured coding framework derived by study aims. Two coders analysed the data, focusing on preference for explanation type, reasons for prompt regeneration, changes in trust and adaptive behaviours. Discussions were transcribed and analysed deductively by two coders. Discrepancies were resolved through discussion rather than formal inter‐coder agreement statistics. Themes were developed by grouping related codes in accordance with Braun and Clarke's method [30].

## 3 Results

### 3.1 Quantitative Results

Quantitative results are presented for failed solutions, time spent solving each problem, explanation regeneration and trust. Across these outcomes, prescriptive explanations were associated with fewer explanation regenerations and higher trust, while differences in failed solutions and task time did not reach statistical significance.

Participants were predominantly female (16:4) with an age range of 20–35 years (mean = 26.9, SD 4.30).

### 3.2 Explanation Types and Trust Results

Figure 1a shows that participants had fewer failed solutions with prescriptive explanations than descriptive explanations, although this difference did not reach statistical significance (paired *t*‐test, *p* = 0.0851; Cohen's *d*_z_ = 0.41). Figure 1b shows a similar pattern for time spent on questions, with lower task times for prescriptive explanations, although the difference was not statistically significant (paired *t*‐test, *p* = 0.0712; Cohen's *d*_z_ = 0.43). Figure 1c shows a statistically significant difference in the number of explanations generated across explanation types (Wilcoxon signed‐rank test, *p* = 0.0006; *r* = −0.84), with prescriptive explanations associated with fewer explanation generations. Descriptive statistics and paired differences are presented in Table 1.

**FIGURE 1:**
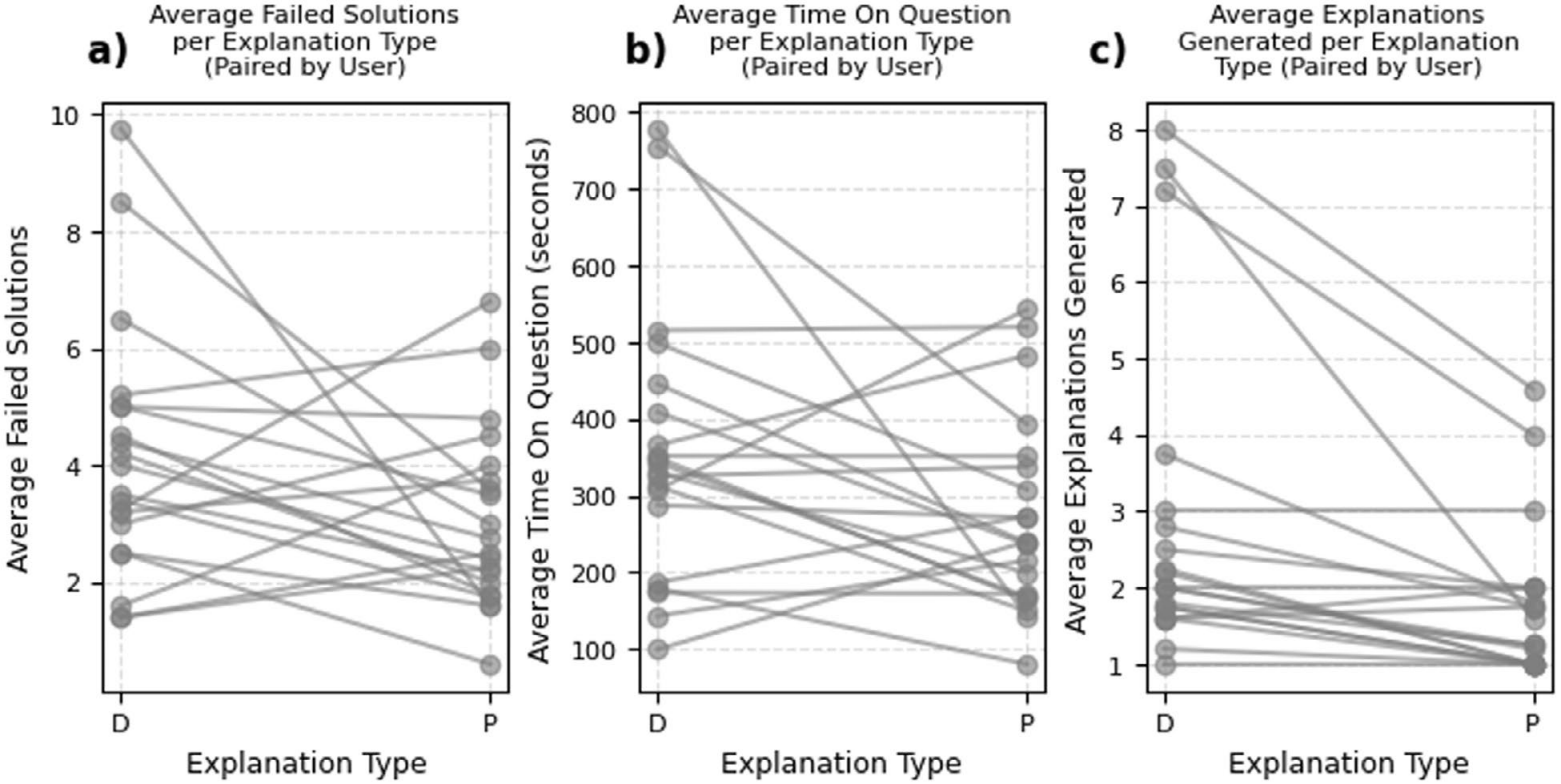
(a–c) Quantitative outcomes by explanation type. (a) Average failed solutions per explanation type paired by user. (b) Average time on question per explanation type paired by user. (c) Average explanations generated per explanation type paired by user.

**TABLE 1:** Descriptive and inferential statistics for paired comparisons between descriptive (*D*) and prescriptive (*P*) explanation conditions.

	*D*	*P*	Difference (*D* – *P*)	Test	Statistic	*p*	Effect size
Figure 1a	4.14 ± 2.17	3.07 ± 1.57	1.07 (95% CI –0.16 to 2.30)	Paired *t*‐test	*t* = 1.817	0.0851	Cohen's *d*_z_ = 0.406
Figure 1b	357.79 ± 179.09	274.41 ± 129.38	83.38 (95% CI –7.92 to 174.68)	Paired t‐test	*t* = 1.911	0.0712	Cohen's *d*_z_ = 0.427
Figure 1c	2.00 (1.71–2.85)	1.43 (1.00–2.00)	0.75	Wilcoxon signed‐rank	*W* = 4.000	0.0006	*r* = −0.843
Figure 2	3.36 (2.80–3.87)	3.62 (3.02–4.01)	−0.20	Wilcoxon signed‐rank	*W* = 37.000	0.0094	*r* = −0.568

*Note:* For normally distributed paired differences, values are reported as mean ± SD and differences as mean paired differences with 95% confidence intervals. For non‐parametric comparisons, values are reported as median (IQR) and differences as median paired differences.

Figure 2a shows the overall trust scores for each user following each explanation type. The Shapiro–Wilk test indicated a deviation from normality; therefore, a Wilcoxon signed‐rank test was used (Table 1). A statistically significant difference was observed (*p* = 0.0094), with a moderate effect size (*r* = −0.57), indicating that prescriptive explanations were associated with higher trust scores.

**FIGURE 2:**
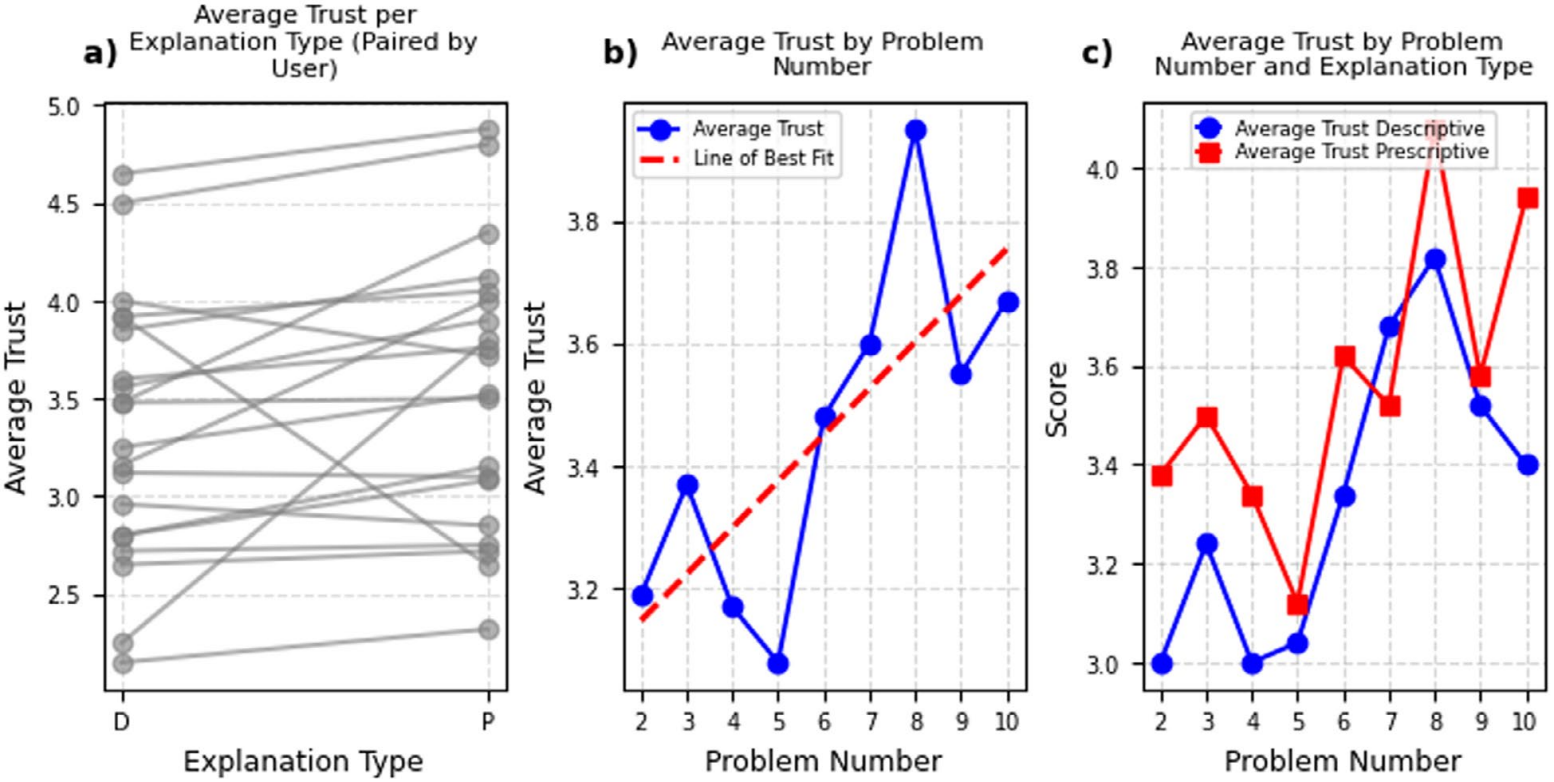
(a–c) Trust quantitative outcomes. (a) Average trust per explanation type (by user). (b) Average trust by problem number. (c) Average trust by problem number and explanation type.

Figure 2b,c show average trust scores across all users by (a) problem number and (b) problem number and explanation type, respectively. Ordinary least squares regression indicated a significant positive association between problem number and trust overall (*β* = 0.076, 95% CI 0.015–0.136, *t* = 2.943, *p* = 0.0216). Similar positive associations were observed for descriptive explanations (*β* = 0.079, 95% CI 0.011–0.146, *t* = 2.757, *p* = 0.0282) and prescriptive explanations (*β* = 0.073, 95% CI 0.001–0.144, *t* = 2.411, *p* = 0.0467); see Table 2.

**TABLE 2:** Ordinary least squares regression results for average trust across problem number, overall and by explanation type.

Analysis	Slope (*β*)	95% CI	*t*	*p*
Overall	0.076	0.015–0.136	2.943	0.0216
Descriptive	0.079	0.011–0.146	2.757	0.0282
Prescriptive	0.073	0.001–0.144	2.411	0.0467

*Note:* Slope (*β*) represents the estimated change in average trust score for each successive problem. Positive values indicate increasing trust across the task sequence. Confidence intervals are 95%.

These findings should be interpreted with caution, as problem number reflects progression through a fixed task sequence. Although explanation type was counterbalanced across participants, increasing trust may also reflect learning, task familiarity or repeated exposure to the system rather than changes attributable to explanation type alone.

### 3.3 Qualitative Results

Comments relating to changes in trust (*n* = 31), XAI type preference (*n* = 54), prompt regeneration (*n* = 20) and adaptive approaches (*n* = 43) are summarised with quotes below.

The qualitative findings provided context for the quantitative results. Participants commonly described prescriptive explanations as faster and easier to use, which is consistent with the lower number of explanation regenerations observed quantitatively. However, participants also reported that inaccurate or unsuitable suggestions reduced trust, illustrating that preference for prescriptive explanations did not necessarily indicate uncritical acceptance of their outputs.

#### 3.3.1 Trust Changes

Along with inaccuracies and hallucinations, Claude's approach of offering multiple solutions, whilst generally preferred, at times reduced trust, as reported in the discussion groups. It was commonly noted among participants that, of the prescriptive outputs, one would usually be wrong, reducing its reliability as a support tool. Participants felt that trust increased as their knowledge of understanding of the tool improved, and when outputs supported fast problem solving (Tables 2 and 3).

**TABLE 3:** Trust changes.

Context	Code	Quote
Examples given when asked about instances where trust decreased	Hallucinations	‘*Maybe one of the first hallucinations, I'm not sure. I was just like, that is because that was the first time that it totally just made something up instead of maybe got one number wrong. So, I definitely felt lower trust, but it didn't change my approach because you have to trust it in order for the thing to work.’—S12*
	Inaccuracy	*‘I felt that I was getting played by some of the options, I was like that's not right, yeah and I just got annoyed. Um, maybe I just did it wrong or whatever but yeah I just don't know if I trust it…. Well. I do trust it, but I think because I got played, I didn't for a moment there, but like, overall it's good*.*’—S19*
	Multiple solutions decreased confidence	*‘Overall, yeah. All the solutions it was giving was like, I don't know if it actually went well, to be honest. Cos, so they gave us multiple solutions, right? But still, it just doesn't, it didn't really work out well for me, actually.’—S16*
Examples given when asked about instances where trust increased	Speed	*‘For me, it was when I was able to do it in, like, less than two minutes.’—S09* *‘It gave me the solution straight away. I have more trust in it. And then the others, when it didn't, I was like, I don't want to use this.’—S06*
	Increased use	*‘But then as it went on, yeah, it just felt easier. Kind of like what [they were] saying, like when you got one right and then the next one was kind of very similar, it was just like, oh, yeah, that's great.’—S06*
	Interpreting the tool	*‘I feel like as time went on, I kind of figured out how it was phrasing things. So, it was more that I was learning how to like interpret it, and so I could trust what it was saying more. Yeah.’—S07*
	Using XAI as an indicator not a guide	*‘and that's when I realised, actually, I'm just going to look at the areas that it's telling me to look at. Not necessarily the specific suggestions. And that's when my trust increased.’—S18* *‘Towards the end, I stopped using it's exact suggestion but just kind of looked at the main areas that it mentioned’—S18*

Regarding comment type, participants vastly preferred prescriptive comments—those that offered the problem and solution. However, descriptive comments were sometimes preferred for basic problems, specifically as knowledge of the system improved. Simplicity and brevity were also noted as beneficial attributes of the descriptive comments (Table 4).

**TABLE 4:** Preference for prescriptive over descriptive outputs.

Context	Code	Quote
Examples given outlining why prescriptive prompts were preferred	Complex problems	*‘I was like flying through at the start, I was like, this is so easy. And then it was like, oh my god, this is a stupid one. Because it was offering me the descriptive ones, not the solution ones, for those two. So, yeah, it's getting frustrating. But then I think it got a bit better after that, at the end.’—S14*
	Speed	*‘I think the ones that give like direct instructions are definitely a lot faster than the ones that's just tell you what's the problem, because then it's easier just to go straight to the problem and fix it.’—S13*
	Multiple options	*‘The suggestions were definitely easier because it usually gives you multiple suggestions. And from that you can kind of tell what actually is clashing’—S04* *‘When it gave you the solution or it gave you like 3 possible solutions and all you need to do is like put one, it just made the job so much easier’—S08*
	Descriptive more challenging	*‘When it gave me just the problems […] It's almost like a quiz. Well, not like a quiz. Like a puzzle, you know?’—S04*
Examples given when discussing benefits of descriptive outputs, including preferable attributes	Solutions were not always reliable	*‘Although sometimes the solutions didn't even work. Which I feel like was a bit sore. […] near the end, it maybe gave me just the [description] but I knew where to look from the previous suggestions’—S18*
	Sufficient for basic problems	*‘If it was a simple solution, the description was enough.’—S06*

When discussing trust development, participants discussed moments where they chose to rely on their own judgement. Participants were critical of the information provided by the tool and decided not to follow the instructions at times. Importantly, our experiment was based on realistic rostering problems, but in solving these problems, participants were hesitant to follow instructions that they thought impacted the hypothetical employee. Table 5 outlines user adaptations.

**TABLE 5:** Adaptive approaches.

Code	Quote
Emergent boundary formation	*‘I think those ones, it took me a while to like try figure out, okay, it's giving me this much information, what can I, from my own knowledge, build on what they're telling me, and how to figure it out.’—S06* *‘I think overall, I trusted about like 80% and I think like, the remaining 20% was my own intuition or own judgement.’—S11* *‘And that's when I was like, okay, maybe the AI, like even if they can give you a solution, it might not be correct’—S13*
Navigating misalignment	*‘[I] tried not to play around too much with the rules, like try to figure out a way to actually solve it’—S01* *‘There were definitely times where, to get it to accept the requirements, I was a bit, oh, […] that's a bit mean to do that to that person, you know?’—S09* *‘I notice that the solutions that they've given is like, it's not making sense. Like as in, it's difficult to achieve in like life’—S13*
Actioning boundaries	*‘It said to increase the amount of days and I was like, that doesn't seem right. So, I just ignored that’—S05* *‘I know for some of mine, like, I noticed that partway through, when it would give me a solution, I just opt for going for like the second or third solution and completely ignore the first one.’—S05*

## 4 Discussion

This study explored trust among 20 participants as they utilised different XAI explanation types to solve rostering problems using rostering software. The results suggest that prescriptive explanations were associated with higher trust and fewer explanation regenerations during short‐term use of the XAI tool. Prescriptive explanations were also associated with lower task times and fewer failed solutions, although these differences were not statistically significant. Qualitative discussion data indicated that some participants associated exceptionally short solve times with increased trust in the XAI tool.

In this study, prescriptive explanations appeared to better satisfy the conditions of trust outlined in the introduction [17], by reducing uncertainty and guiding users towards actionable solutions. However, trust decreased when outputs were inaccurate or repetitive. In these cases, participants shifted from reliance to critical evaluation, using the explanation as a guide rather than following it blindly. Overall, qualitative data demonstrated that participants consistently preferred prescriptive explanations. These were perceived as more effective and trustworthy and participants stated in discussion groups that they were faster, although the quantitative data did not support this objectively. Automation bias and over trust in XAI features are well‐documented concerns in the literature [13, 31, 32]. Several studies show that providing explanations does not necessarily reduce automation bias and can, in some cases, increase overreliance on AI recommendations [32, 33]. Our qualitative data suggest that participants employed critical judgement early in their problem solving which caused them to hesitate before acting on the tool's recommendation, particularly when the solution suggested seemed unfair to a hypothetical staff member. This may have been influenced by the realistic nature of the rostering simulation, which included staff names, duties and departments. These human details may have made the task feel authentic enough to trigger ethical consideration, even though participants knew it was a simulation. This echoes evidence from vignette studies where realistic, person‐centred scenarios prompt ethically engaged decision making [34] and promotes empathy [35]. Our findings suggest that incorporating human characters and contextual details in XAI‐supported tasks may encourage users to consider the human impact of AI‐generated recommendations. Whether this influences automation bias is an area of further investigation.

Furthermore, our results differ from the pattern suggested by Hoffman et al.'s proposed notional view of trust morphology [16]. Our participants demonstrated progressive trust development over time with a lower initial peak than suggested. These results indicate that, for students, trust in XAI appears to progress more consistently upwards, lacking the early peak and trough suggested in the Hoffman et al. model [16]. This aligns with previous findings, whereby higher trust in AI has been identified among young adults [36]. Moreover, close alignment of trust in the early stages of the experiment provides insight, balanced expectations likely explain the steadier trust development and the avoidance of the upwards surge attributed to the experience of ‘surprise’. Qualitative data expanded these findings, demonstrating sensitivity to inaccuracies and the early and ongoing development of critical boundary formation throughout XAI use. These findings suggest that participants developed increasing trust alongside evidence of critical engagement with XAI outputs. Further research is needed to determine whether this pattern is observed among experienced healthcare professionals.

### 4.1 Limitations

The small sample size and narrow age‐range limit the generalisability of the findings, as more experienced clinicians may demonstrate different trust dynamics, mental models and preferences. Participants were final‐year medical imaging students, which may further limit applicability to other healthcare populations.

There was no control condition without XAI, limiting the ability to isolate the effect of XAI on outcomes such as trust over time. Although explanation type order was counterbalanced across participants, the rostering problems were presented in a fixed sequence. Consequently, learning, task familiarity and repeated exposure to the system may have influenced responses over time, and these effects cannot be completely separated from explanation type.

The utilised trust scale was not independently validated, and its reliability was inferred from related scales; this limits the validity and generalisability of the study findings.

The XAI explanations did not adapt in real time to user behaviour, which at times caused confusion among participants. This reflects the prototype nature of the system, meaning the findings may not fully transfer to higher‐fidelity tools that dynamically respond to user input.

The XAI feature used a single LLM, and outputs were not independently checked for factual accuracy after generation. Although output variability was part of the interaction being studied, variability between generations was not independently quantified. These factors may limit the reproducibility of specific outputs and should be considered when interpreting findings from this prototype system.

Trust was measured using self‐reported scales, which are susceptible to response bias. Researchers were present during the experiment to provide technical support, which may have introduced unintended influence on participant behaviour.

### 4.2 Future Implications

For this reason, future research should include greater cohort sizes and longer time frames. Including experienced clinicians would demonstrate whether preferences observed in this study generalise to higher levels of domain expertise. Further research could also explore the impact of humanisation on trust calibration and its relationship with automation bias for healthcare technologies.

It remains unclear as to whether adaptive XAI systems that dynamically shift between descriptive and prescriptive explanation modes based on user preference and task complexity may be preferable in some settings. For low‐resource AI systems, highlighting areas of concern visually may be more effective than providing explicit task instructions.

Future studies should include larger samples, experienced clinical users and randomised or counterbalanced task sequences. Comparison with a condition without XAI would also help determine whether changes in trust or performance are attributable to the presence of explanations rather than repeated exposure to the task. Evaluation in real‐world rostering environments is needed to determine whether the observed preferences and behaviours translate beyond the simulated setting.

## 5 Conclusion

This study utilised human‐grounded evaluation to explore trust development among medical imaging students. Participants used XAI to solve rostering problems and recorded their trust on a modified scale after each problem was either solved or abandoned. Prescriptive outputs were strongly preferred over descriptive ones and were associated with fewer explanation regenerations and higher overall trust. Participants also demonstrated critical engagement with AI‐generated outputs, identifying inaccuracies and adapting how they used the explanations. These findings suggest that explanation type influences how novice users interact with XAI in a simulated medical imaging rostering task, but further research with experienced clinical users is required to determine whether these findings translate to clinical practice.

## Funding

This study was supported by Unitec's Early Career Researcher's Grant.

## Ethics Statement

Ethics approval for this study was obtained from the Unitec Research Ethics Committee (UREC) (Application number 2024‐1049).

## Consent

Informed consent was obtained from all participants.

## Conflicts of Interest

The authors declare no conflicts of interest.

## Supporting information


**Appendix A:** Prompt Template.

**Appendix B:** See Figures B1 and B2.

## Data Availability

The data that support the findings of this study are available from the corresponding author upon reasonable request.
