# Supplementary material for: Describe or Direct? Artificial Intelligence Explanation Type, Trust and User Behaviour in a Medical Imaging Rostering Task
Source: J Med Radiat Sci. 2026 Oct 20:10.1002/jmrs.70137. Online ahead of print. doi: 10.1002/jmrs.70137 (PMC13683881; doi:10.1002/jmrs.70137)
Supplement: Supplementary file 1 [file JMRS-9999-0-s001.docx]

### Appendix A

### Prompt Template

All prompts followed a consistent structure comprising a static context segment, a scenario-specific problem statement, and an explanation instruction.

#### Static Context Segment

This employee has a conflict in the roster model and cannot be generated. This occurs when there is a contradiction between rules, available allocations, and fixed allocations. Provide a short, basic explanation of why this is happening and potential ways to solve it like changing rule values. Don't reiterate any data from the prompt. Use numbers if needed. Use simple language that is easily understood by someone who is not a rosterer or scheduler. Use language that can be understood by someone who did not complete high school level education. Avoid the words 'allocation', 'conflict', 'constraint', 'model' and similar technical language. 'Fortnight' is an acceptable term to use. Provide only solutions that are realistic. Do not refer to talking to the boss, and do not refer to yourself. An employee can only start one shift per day (or have a day off) although night shifts can cross to next day. If an exact conflict resolution isn't clear, simply state that it is indeterminate based on the given constraints. Rules restrictions(optional) are either Maximum, Minimum or Equal. Equal means exactly that many, e.g. Equal for hours means an exact number of hours. Also suggest maybe changing one of the rule values (just say higher or lower). Our model goes that every employee has an onstretch followed by an offstretch followed by an onstretch and so forth. Days on/hours/shifts in a row rules affect onstretches, days off in a row rules affect offstretches.

#### Scenario Example

There is a rule conflict between the following sets of rules: rule set 1: 'Min days off after a stretch of nights' of 3, rule set 2: 'Hours per 2 weeks' of 80, rule set 3: 'Min weekends off per rolling three weeks' of 1, 'Max 2330 GEN shifts per roster' of 5. If any of these rule sets are deleted, the roster will solve.. If the following allocations are deleted, the roster will solve (without deleting any rules): Shift 2330 GEN on Monday 24/02/2025, Shift 2330 GEN on Sunday 23/02/2025, Shift 2330 GEN on Saturday 22/02/2025. Here is a list of all shifts an employee can work: 0700 ED (from 7:00 AM to 3:30 PM, hours: 8), 0830 ED (from 8:30 AM to 5:00 PM, hours: 8), 0800 GEN (from 8:00 AM to 4:30 PM, hours: 8), 0830 GEN (from 8:30 AM to 5:00 PM, hours: 8), 1230 Late (from 12:30 PM to 9:00 PM, hours: 8), 1430 Late (from 2:30 PM to 11:00 PM, hours: 8), 0800 W/E (from 8:00 AM to 4:30 PM, hours: 8), 1500 W/E (from 3:00 PM to 11:00 PM, hours: 8), 0730 CT (from 7:00 AM to 3:30 PM, hours: 8), 0800 CT (from 8:00 AM to 4:30 PM, hours: 8), 1300 CT (from 1:00 PM to 9:00 PM, hours: 8), 0800 CT W/E (from 8:00 AM to 4:00 PM, hours: 8), 0700 MRI (from 7:00 AM to 3:30 PM, hours: 8), 1230 MRI (from 12:30 PM to 9:00 PM, hours: 8), 2330 GEN (from 11:30 PM to 7:30 AM, hours: 8). Here is a list of all tasks an employee can work: . The roster starts on 10/02/2025 12:00:00 AM. It is 56 days total.

Descriptive condition:
Describe the issue in 1-2 concise sentences. Do not provide solutions. Avoid all technical language

Prescriptive condition:
Explain the issue in 1-2 concise sentences. Provide concise solutions. Fortnightly hours are unlikely to be the issue. Avoid all technical language

#### Descriptive Output Example

The worker must work 80 hours in 2 weeks but can't meet this due to night shift rules. After night shifts, they need 3 days off, but with weekend limits and a max of 5 night shifts, there aren't enough other days left to get to 80 hours.

#### Prescriptive Output Example

The system can't make a schedule because the night shifts on Feb 22-24 clash with rules about weekend time off, recovery days after nights, and total night shifts allowed.

Possible fixes:

Move some of the night shifts to different days

Lower the minimum recovery days after nights from 3 to 2

Let the person work 6 night shifts instead of 5 per roster

Remove one of the night shifts on Feb 22-24

#### Additional Notes

All prompts used the same static structure, with only the scenario content and final instruction varying between conditions. Regenerated outputs were produced using identical prompts and were not modified prior to presentation.
