# Supplementary material for: Describe or Direct? Artificial Intelligence Explanation Type, Trust and User Behaviour in a Medical Imaging Rostering Task
Source: J Med Radiat Sci. 2026 Oct 20:10.1002/jmrs.70137. Online ahead of print. doi: 10.1002/jmrs.70137 (PMC13683881; doi:10.1002/jmrs.70137)
Supplement: Supplementary file 2 [file JMRS-9999-0-s002.docx]

## Appendix B

Appendix B Figure 1: Experimental task and explanation-type sequence.

S01–S20 represent participant identifying numbers. The rows represent the 20 rostering problems, which were completed by all participants in the same fixed sequence. Green indicates descriptive explanations and blue indicates prescriptive explanations. Explanation type was counterbalanced across participants: S01–S10 received descriptive explanations for odd-numbered problems and prescriptive explanations for even-numbered problems, while S11–S20 received the reverse sequence. Rostering problems were given fictional New Zealand names for the purpose of the experiment.

Appendix B Figure 2: Experimental setup and rostering task environment.

(a) Example participant worksheet used during the experiment, showing task instructions, explanation display area, and post-task trust survey section (survey link removed). Participants generated AI explanations externally using Claude and attempted to resolve the rostering issue using the provided explanation.
(b) Rosterlab interface displaying an example unresolved rostering problem used within the simulated workflow task. Participants interacted with the rostering system to identify and resolve scheduling conflicts.
